# HTT-DB: new features and updates

**DOI:** 10.1093/database/bax102

**Published:** 2018-01-04

**Authors:** Bruno Reis Dotto, Evelise Leis Carvalho, Alexandre Freitas da Silva, Filipe Zimmer Dezordi, Paulo Marcos Pinto, Tulio de Lima Campos, Antonio Mauro Rezende, Gabriel da Luz Wallau

**Affiliations:** 1 Campus São Gabriel, Universidade Federal do Pampa, Avenida Antonio Trilha, 1847, São Gabriel, Rio Grande do Sul, 97300-000; 2 Pos Graduation in Biological Sciences, Universidade Federal do Pampa, Avenida Antonio Trilha, 1847, São Gabriel, Rio Grande do Sul, 97300-000; 3 Pos Graduation in Biosciences and Health Biotechnology, Aggeu Magalhães Institute (IAM), Avenida Professor Moraes Rego, s/n, Recife, Pernambuco, 50740-465; 4 Bioinformatic Core, Aggeu Magalhães Institute (IAM), Avenida Professor Moraes Rego, s/n, Recife, Pernambuco, 50740-465; 5 Department of Microbiology, Aggeu Magalhães Institute (IAM), Avenida Professor Moraes Rego, s/n, Recife, Pernambuco, 50740-465; 6 Department of Entomology, Aggeu Magalhães Institute (IAM), Avenida Professor Moraes Rego, s/n, Recife, Pernambuco, 50740-465

## Abstract

Horizontal Transfer (HT) of genetic material between species is a common phenomenon among Bacteria and Archaea species and several databases are available for information retrieval and data mining. However, little attention has been given to this phenomenon among eukaryotic species mainly due to the lower proportion of these events. In the last years, a vertiginous amount of new HT events involving eukaryotic species was reported in the literature, highlighting the need of a common repository to keep the scientific community up to date and describe overall trends. Recently, we published the first HT database focused on HT of transposable elements among eukaryotes: the Horizontal Transposon Transfer DataBase: Database URL: (http://lpa.saogabriel.unipampa.edu.br: 8080/httdatabase/). Here, we present new features and updates of this unique database: (i) its expansion to include virus-host exchange of genetic material, which we called Horizontal Virus Transfer (HVT) and (ii) the availability of a web server for HT detection, where we implemented the online version of vertical and horizontal inheritance consistence analysis (VHICA), an R package developed for HT detection. These improvements will help researchers to navigate through known HVT cases, take data-informed decision and export figures based on keywords searches. Moreover, the availability of the VHICA as an online tool will make this software easily reachable even for researchers with no or little computation knowledge as well as foster our capability to detect new HT events in a wide variety of taxa.

**Database URL**: http://lpa.saogabriel.unipampa.edu.br:8080/httdatabase/

## Introduction

Genetic inheritance is the main mode of genetic material transmission from ancestral to descendent individuals or species. Such process is widely known as vertical transfer of genetic material. However, there is another phenomenon which allows the transfer of genetic material between biological entities known as Horizontal Transfer (HT). Such HT events are very frequent in prokaryotic species, having a great impact on the exchange of different classes of genes such as anti-biotic resistance genes. However, in recent years, investigation of HT events frequency and their impact in multi-cellular eukaryotic genomes have been underscored (1).

Historically HTs are named based on the genetic entity which is transferred between species as Horizontal Gene Transfer (HGT) and Horizontal Transposon Transfer (HTT). HGT is a rare phenomenon among eukaryotes and few examples are known, taking place in particular conditions such as host-parasite relationships (2). HTT is a much more frequent phenomenon among eukaryotes in which transposable elements (TEs), DNA fragments that are capable of moving themselves between different genomic positions, transfer from one host species to another by means other than sexual reproduction (3).

Most of the knowledge about HT events is dispersed on original research articles, but some effort has been made to compile such information, at least for HGT events among Bacteria and Archaea (4, 5). However, no database was available for HT events among eukaryotic species until 2015. Based on this gap, we developed the Horizontally Transferred Transposable Element Database (HTT-DB) which allows researchers to have access to all known HTT cases among eukaryotes and perform searches on these data using TEs [Repbase––(6) and host species (https://www.ncbi.nlm.nih.gov/taxonomy)] keywords (7). To date, our database was cited in 7 other publications and had >1000 page views since its publication. Moreover, 2276 new HTT cases were added to the database since its publication covering a wide variety of taxa (**Cumulative number of HTT in the last 10 years** panel–http://lpa.saogabriel.unipampa.edu.br:8080/httdatabase/).

Here, we present HTT-DB’s new features and updates which make it richer in details/information regarding HT of genetic material between viruses and host (Host-Virus Transfer–HVT) as well as the availability of a web server implementing a recently published method for HT detection (8).

## Database interface for horizontal virus transfer

HT of genetic material between viruses and their hosts is another phenomenon not fully appreciated so far. Nevertheless, in the last years, accumulating evidence has shown that this exchange is more common than previously recognized (9–11). Such events are also known as endogenization, and the viral remains, found integrated in the host genomes, are commonly called endogenous viral elements (EVEs) (12–14). Due to the absence of a database for such events which can have huge impact on understanding of genome biology of host species (15, 16), we decided to expand the HTT database to include such endogenization events in the same fashion as HTT events. Here, we call such events Horizontal Virus Transfer (HVT) in order to emphasize the genetic entities that are being transferred: the viral sequences. The main difference of HVT compared to HTT is the database searching that can be performed with viral classification scheme established by the International Committee on Taxonomy of Virus (ICTV–https://talk.ictvonline.org/) instead of the TEs Repbase classification. Moreover, database navigation and image generation depending on the user selected keywords follow the same rationale already implemented in HTT-DB. Such new database layer will be updated annually with new published information obtained from manuscripts recovered from PubMed-NCBI and Google Scholar searchers with the following terms ‘endogenous virus,’ ‘EVEs,’ ‘endogenization’ and ‘viral derived elements.’ Furthermore, any user can contribute sending new data to the database through the ‘Add new data–HVT database (Virus)’ menu directly through the system or downloading an example table. The user also can submit nucleotide sequences of TEs and endogenous virus which will be available along with its corresponding metadata to download.

**Figure 1.**
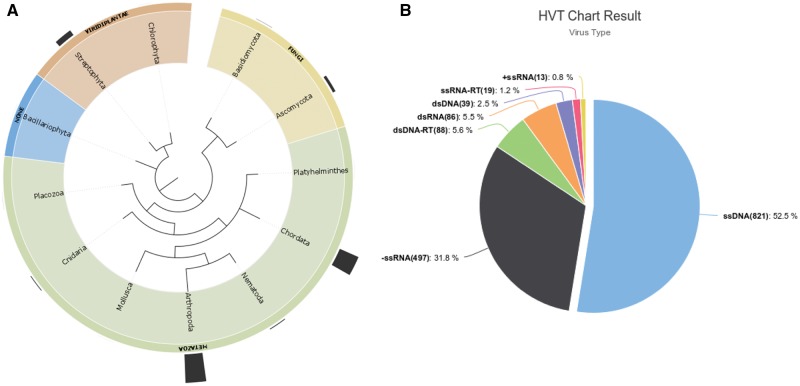
(**A**) Distribution of HVT events (endogenization) across different Phylum, outermost bars represent the number of HVT cases in the taxa shown. (**B**) HVT chart result from database search including all cases reported in the database, ssDNAs >50% of all known HVT events.

Based on our curation of HVT events reported in the literature and added to the HTT-DB we found a total of 1563 HVT events with the majority of cases reported in Metazoan genomes (1279 endogenization events), and a high proportion of cases found in the Phylum Arthropoda (721) and Chordata (516) (Figure 1A). Exploring the viral classification scheme from the ICTVs, we can observe that the largest amount of endogenization events is accounted by the single strand DNA viruses (ssDNA—821) followed by negative single strand RNA viruses (-ssRNA—497) (Figure 1B). Deeper exploration at lower taxonomic levels can be performed by the users both at the host and viral taxa following the drop down menus of the ‘Database search button.’

## Interface for vertical and horizontal inheritance consistence analysis

**Figure 2.**
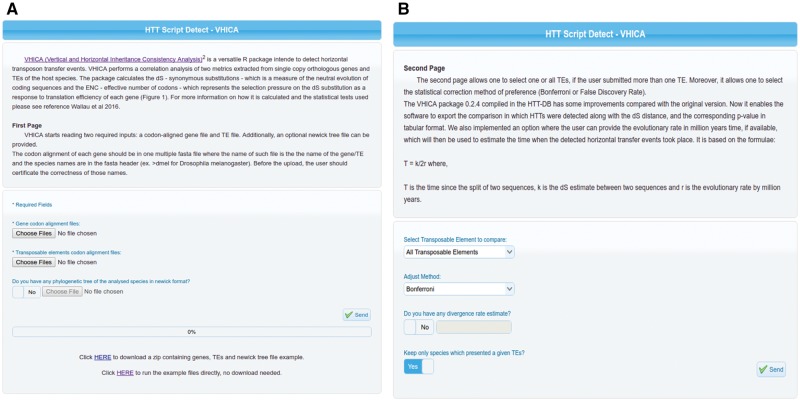
(**A**) First panel of VHICA interface available for HTT detection through the HTT-DB database. Two optional (Gene Files and TE Files) and one optional box (Phylotree File) for files upload are available as well as an example of all those files used in the original VHICA paper (Wallau *et al.* 2016). (**B**) Second panel of VHICA interface. Here, the user can select one, a set of or all TEs for run VHICA. Since VHICA performs multiple comparisons there are two correction methods available for the user choice: Bonferroni and False Discovery Rate. TE divergence rate can be added, and then VHICA will estimate the HT time in Mya. User can also choose if they want to plot the results only for hosts’ species which presented a given TE or maintain all the other species in the plotting step.

Vertical and Horizontal Inheritance Consistence Analysis (VHICA) is an R package developed to detect HT events (8). This package implements several command line R functions capable of reading codon aligned host and TEs sequences, extracting codon bias and synonymous substitutions and performing statistical analysis on these extracted parameters in order to evaluate the presence of HT signal. Although bioinformatics curriculum is being implemented in a growing number of under-graduate courses in biological sciences, several biologists and scientists from associated areas still have difficulties facing command line software (17–19). In order to overcome such issue and make VHICA available to a wide variety of researchers, we implemented it as a web server in the HTT-DB. As can be seen in first panel of VHICA, there are three fields where the user can upload the input data (Figure 2A). Besides, in the same fields, one can have access to example files. Example files are the full dataset used by Wallau *et al*. 2016 which the user can apply as input in order to see the VHICA output or check the alignments and gene/TE names formats needed to run VHICA properly. In addition, we also added detailed information regarding file formats and VHICA analysis in the ‘**About**’ Section ‘**VHICA Online–Vertical and Horizontal Inheritance Consistency Analysis (VHICA package)**’ sub-section of HTT-DB. In the second panel, the user can choose a specific TE or all TEs to be used and tested in VHICA, the multiple-testing correction method, and an optional field: the divergence rate. The last option is a new VHICA feature which allows an estimation of the time when the HT event took place using the equation *T = k/2r* (20) (Figure 2B)*. T* represents the divergence time between TEs, *k* is the synonymous divergence (dS) between TEs and *r* is the TE specific evolutionary rate.

**Figure 3.**
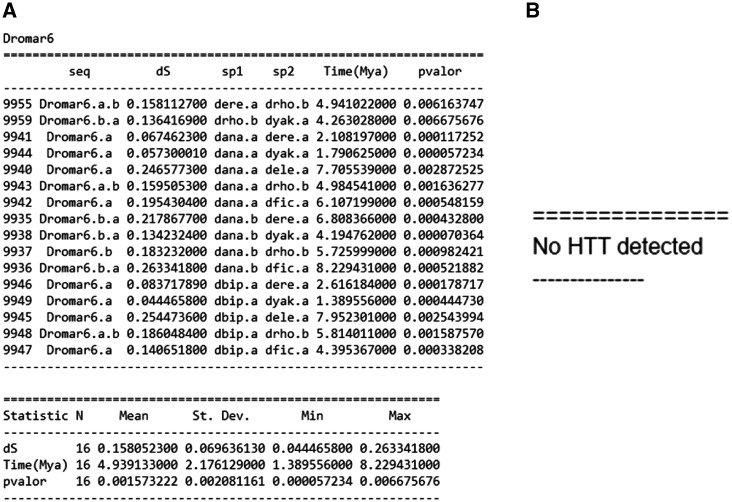
Three possible VHICA outputs. (**A**) Summary of all statistically supported HTT cases and associated data. (**B**) Resulting output when no significant HTT signal is detected. (**C**) Graphical output showing all host species and the significant pairwise comparison following *P*-value the legend colour.

VHICA can output two types of results: (i) if all TEs were selected for analysis, it will report a summary of all statistically significant pairwise comparison as well as the dS, the pair of species involved, the estimated HT time in Mya and the associated *P*-value or a text file with ‘No HTT detected message’ (Figure 3A and B). (ii) A plot in .pdf format containing the *P*-value matrix for all TEs evaluated (Figure 3C). All these results become available as a compressed .zip file that will remain stored in the server and available for download for 10 days.

## Conclusion

In summary, we present here the expansion of the only available database about HT among eukaryotic species. Currently, HTT-DB expansion includes HVT (endogenization of viral sequences into the host genomes) and we made available a R package (VHICA) developed to detect HT events through the HTT-DB interface.

*Conflict of interest*. None declared.
